# Evaluating algorithms for identifying incident Guillain-Barré Syndrome in Medicare fee-for-service claims

**DOI:** 10.1016/j.gloepi.2024.100145

**Published:** 2024-05-03

**Authors:** Samantha R. Eiffert, Brad Wright, Joshua Nardin, James F. Howard, Rebecca Traub

**Affiliations:** a Division of Pharmaceutical Outcomes and Policy, University of North Carolina School of Pharmacy, Chapel Hill, NC, USA; b Health Services Policy and Management, Arnold School of Public Health, University of South Carolina, Columbia, SC, USA; c Department of Neurology, University of North Carolina School of Medicine, Chapel Hill, NC, USA

**Keywords:** Guillain-Barré syndrome, GBS, Algorithm, Validation, Medicare, Chart review

## Abstract

**Objective:**

Claims data can be leveraged to study rare diseases such as Guillain-Barré Syndrome (GBS), a neurological autoimmune condition. It is difficult to accurately measure and distinguish true cases of disease with claims without a validated algorithm. Our objective was to identify the best-performing algorithm for identifying incident GBS cases in Medicare fee-for-service claims data using chart reviews as the gold standard.

**Study design and setting:**

This was a multi-center, single institution cohort study from 2015 to 2019 that used Medicare-linked electronic health record (EHR) data. We identified 211 patients with a GBS diagnosis code in any position of an inpatient or outpatient claim in Medicare that also had a record of GBS in their electronic medical record. We reported the positive predictive value (PPV = number of true GBS cases/total number of GBS cases identified by the algorithm) for each algorithm tested. We also tested algorithms using several prevalence assumptions for false negative GBS cases and calculated a ranked sum for each algorithm's performance.

**Results:**

We found that 40 patients out of 211 had a true case of GBS. Algorithm 17, a GBS diagnosis in the primary position of an inpatient claim and a diagnostic procedure within 45 days of the inpatient admission date, had the highest PPV (PPV = 81.6%, 95% CI (69.3, 93.9). Across three prevalence assumptions, Algorithm 15, a GBS diagnosis in the primary position of an inpatient claim, was favored (PPV = 79.5%, 95% CI (67.6, 91.5).

**Conclusions:**

Our findings demonstrate that patients with incident GBS can be accurately identified in Medicare claims with a chart-validated algorithm. Using large-scale administrative data to study GBS offers significant advantages over case reports and patient repositories with self-reported data, and may be a potential strategy for the study of other rare diseases.

## Highlights

• Guillain-Barré Syndrome (GBS) is rare and claims data offer larger sample sizes.
• Tested algorithms for identifying GBS in claims with chart review gold standard.
• Primary inpatient diagnosis + procedure had the highest positive predictive value.
• Primary inpatient diagnosis was favored across three GBS prevalence assumptions.
• We validated several algorithms for identifying GBS cases in Medicare claims.

## Background

Guillain-Barré Syndrome (GBS) is a rare, acute, immune-mediated neuropathy that can occur following respiratory or gastrointestinal infections (e.g., *Campylobacter jejuni*, influenza, Zika virus, and others), vaccination, surgical procedures, and checkpoint inhibitor chemotherapy. [[1], [2], [3], [4], [5], [6], [7], [8], [9], [10]] GBS is characterized by an acute onset, monophasic, rapidly progressive polyneuropathy that may result in respiratory failure requiring mechanical ventilation in approximately 30% of cases. It has an estimated yearly incidence of 1–2 cases per 100,000 people. [11] While routinely collected, large-scale administrative data (e.g., health insurance claims) have tremendous potential to advance the study of rare diseases, accurately identifying patients with the exposure or disease of interest is important because these data sources are primarily used for reimbursement—rather than clinical—purposes. Using a validated algorithm to identify a patient population is important because the validation study demonstrates the extent of misclassification compared to a gold standard. [12,13]

There is one available validation study for identifying patients with GBS in claims data, but this was restricted to adolescents age 11 through 21 with commercial insurance. The authors found that an ICD-9-CM diagnosis code for GBS in the primary position for inpatient claims compared to chart review as the gold standard resulted in a positive predictive value (PPV) of 56%. They were able to achieve a PPV of 70% (95% CI: 59–80%) when requiring the ICD-9-CM code to appear in the primary position of an inpatient claim and any claim associated with a neurologist visit at any point during the follow-up period. [14] Beyond this, several vaccine studies have reported the PPV of a GBS diagnosis code compared to chart review, but no study has validated a series of algorithms for identifying older adults with GBS in Medicare claims.

The studies in Medicare that report the PPV of a GBS diagnosis code used either ICD-9-CM or ICD-10-CM diagnosis codes with various position requirements. One study used the ICD-10-CM diagnosis code for GBS in the primary position of an inpatient claim and reported a PPV of 71.2%. [15] Another study used the ICD-9-CM diagnosis code for an inpatient claim with no position requirement and found a PPV of 64.5%. [16] Two additional studies found a PPV of 68.2% [17] and 82% [18] when the ICD-9-CM code was in the primary or secondary position for an inpatient claim.

We identified two additional studies that identified GBS patients in claims. One used an ICD-9-CM GBS diagnosis code in inpatient claim in Kaiser Permanente data and reported a PPV of 61.4%. [19] The other study used a non-validated algorithm in the Merative MarketScan® Commercial Claims and Encounters Database from 2009 to 2015 identified patients by requiring a GBS diagnosis code in the primary position for an inpatient claim and at least one inpatient claim for any of the following procedures: lumbar puncture, electromyography, or nerve conduction study within 45 days of the GBS index diagnosis date. [20]

We developed and tested a series of algorithms that included both ICD-9-CM and ICD-10-CM GBS diagnosis codes and assessed the impact on PPV when changing the position of the code, requirements for codes originating from inpatient or outpatient settings, and including other variables such as diagnostic studies or following up with a neurologist. Our goal was to identify the best-performing algorithm for identifying incident GBS cases in Medicare fee-for-service claims data using chart reviews as the gold standard. Importantly, this study is not testing diagnostic algorithms, but rather is intended to be used by researchers interested in identifying GBS cases in claims data.

## Methods

### Data and study subjects

We used Medicare claims data linked to electronic health records at the University of North Carolina Health System (UNCH) to conduct a retrospective study to identify incident cases of GBS. To be part of the Medicare-linked sample, a Medicare beneficiary must have one or more visits to any hospital in the UNC Health System across North Carolina. The Carolina Data Warehouse for Health stores UNC electronic health record (EHR) data, and Medicare claims are available for patients treated at a UNC facility from 2015 to 2019. Using this Medicare linkage sample, we identified patients with an inpatient or outpatient GBS diagnosis code (ICD-9-CM 357.0 and ICD-10-CM G61.0) in any position from 2015 to 2019 with Medicare Parts A and B enrollment at the time of the diagnosis. We identified all patients with a diagnosis code for GBS in any position for either an inpatient or outpatient claim.

### Chart reviews

We requested EHR information from the North Carolina Translational and Clinical Sciences Institute for patients we identified in the Medicare-linked sample with a diagnosis code for GBS. Not all patients with a GBS diagnosis code in the Medicare-linked sample have an associated medical record at UNC with a GBS diagnosis. This occurs when patients are treated outside of UNCH, and these patients were excluded since we considered medical records to be the gold standard and there was no way to verify these patients were true cases. We uploaded the patient list of those with a GBS diagnosis in their medical record (e.g., GBS was treated or followed-up with at UNCH) into a REDCap database where three neurologists conducted chart reviews, determined whether the patient was a true GBS case, and assigned Brighton Criteria to indicate diagnostic certainty (i.e., Level 1 = most certain, Level 4 = least certain) based on review of the medical records. In instances where there was uncertainty regarding whether a patient was a true case or to which level of diagnostic certainty the patient belonged, the three neurologists discussed and reached a consensus. The Brighton Criteria are based on the presence or absence of specific symptoms including weakness of limbs, decrease or loss of reflexes, time from onset to nadir of 12 hours to 28 days, and test results from spinal tap and nerve conduction studies. [21]

### Linkage

We exported the chart reviews from REDCap into a SAS dataset that we linked to Medicare claims from 2015 to 2019. We allowed a +/− 6-day window for the Medicare claim diagnosis date to be considered aligned with the diagnosis date in the EHR. If the GBS diagnosis occurred during a hospitalization, we assigned the GBS diagnosis date to be the date of the inpatient admission for GBS.

### Algorithms

We reviewed the literature on GBS as well as other rare conditions to identify, adapt, and develop several algorithms that rely on different elements contained in Medicare claims including diagnosis codes, procedure codes, and provider types. As shown in Table 2, we considered different combinations of these elements, as well as whether the data were pulled from inpatient or outpatient claims, and whether the diagnosis was in the primary or other position on the claim. We then tested each of these algorithms against our chart-confirmed GBS population.

### Data analysis

We calculated and reported the positive predictive value (PPV) for each algorithm using the PPV-specific standard error formula to calculate 95% confidence intervals. [22] PPV is the percentage of potential GBS cases identified in claims data that were confirmed as true cases during chart review. [14] While PPV is dependent on the prevalence of the disease in the population, reporting other measures of algorithm performance (e.g., sensitivity, specificity, and negative predictive value) would have required chart review of a random sample of cases not flagged as potential GBS cases in the claims. [14,23] Given the rarity of GBS, it is likely that an extremely large number (e.g., millions) of charts would need to be reviewed to find a single case of GBS in the EHR for which there is no evidence of GBS in the claims data. Given the time and resources involved in conducting chart reviews, such an approach was not feasible. To address this limitation, we conducted several robustness checks using different hypothetical samples of claims without evidence of GBS and applying different assumptions about the prevalence of true GBS cases in that group (i.e., false negatives). The three scenarios we tested were: no false negatives, the same rate of false negatives as true positives, and false negatives equal to the overall prevalence of GBS reported in prior literature. We ranked each algorithm's performance on the measures of sensitivity, specificity, PPV, and negative predictive value (NPV). Each algorithm received a score for each measure, from 1 to 18, with 1 representing the best performing algorithm and 18 representing the worst performing algorithm, with ties allowed. We then summed the ranking for each algorithm across the four measures, such that the best possible score would equal 4 (i.e., 1 × 4) and the worst possible score would equal 72 (i.e., 18 × 4). To create relative values for easier comparison, we divided each algorithm value by the best possible score for each assumption. We overlayed a heat map with darker green indicting better performing algorithms and red indicating worse performing algorithms. Statistical analyses were performed using SAS 9.1 (SAS Institute Inc., Cary, NC, USA).

## Results

We identified 406 patients with a GBS diagnosis code in any position of an inpatient or outpatient claim in the Medicare-linked sample with Parts A and B enrollment at the time of the GBS diagnosis. From this list, there were 211 patients where the GBS diagnosis occurred at a UNCH facility. The cohort of patients with a chart review-confirmed GBS diagnosis was considered the gold-standard (Table 1). There were 40 (19%) true positive incident GBS cases identified via chart review and 171 (81%) false positives. The true positive cases tended to be slightly older than false positive cases. Among true positives 55.0% were female, and among false positives 51.5% were female. There were more true positive cases from 2017 to 2019 than from 2015 to 2016 (60% vs 40%). Among true positive cases, most patients had Brighton Criteria Level 1 or 2, with 32.5% having Level 1 certainty. Among false positive cases, 50.3% were diagnosed from 2017 to 2019. For both groups, the majority of patients were non-Hispanic white, measured using the RTI race variable. [24]

**Table 1:** Demographics of patients included in study.

	True case of GBS		Non-case of GBS	
	*N* = 40	Percent	*N* = 171	Percent
Age				
<70	13	32.5	98	57.3
70–74	13	32.5	27	15.8
≥75	14	35.0	46	26.9
Sex				
Male	18	45.0	83	48.5
Female	22	55.0	88	51.5
Race				
Non-Hispanic white^a^	>29	>72.5	133	77.8
Year of diagnosis				
2015–2016	16	40.0	85	49.7
2017–2019	24	60.0	86	50.3
Brighton Criteria				
Level 1	13	32.5		

a Cell size suppressed according to CMS policy.

The performance results for each of our 18 different algorithms for identifying incident cases of a GBS diagnosis in claims and shown in Table 2. Algorithm 17, a GBS diagnosis in the primary position of an inpatient claim and a diagnostic procedure within 45 days of the inpatient admission date, had the highest PPV of 81.6%. This was closely followed by Algorithm 18 with a PPV of 80.6%, which required a GBS diagnosis in the primary position of an inpatient claim, a neurologist visit and a diagnostic procedure for GBS. Algorithm 15, a GBS diagnosis in the primary position of an inpatient claim, had a PPV of 79.5%. Algorithm 1, any GBS diagnosis code, which was used to identify the study population, had a PPV of 19.0%.

**Table 2:** Positive Predictive Values (PPVs) of algorithms for identifying incident cases of GBS in Medicare claims.

*N* = 211 cases with GBS dx any position of any claim, w/ AB enrollment	GBS	Non-GBS	PPV (%) 95% CI
***Diagnosis codes in any position of inpatient or outpatient claims***			
1. Any GBS diagnosis (in any position of an inpatient or outpatient claim)	40	171	19.0% (13.7, 24.2)
2. Two or more instances of any GBS diagnosis within 30 days [26]	26	35	42.6% (30.2, 55.0)
3. Any GBS diagnosis and a neurologist encounter within 45 days of the diagnosis [14]	37	42	46.8% (35.8, 57.8)
4. Any GBS diagnosis and a diagnostic procedure within 45 days of the diagnosis [20]	34	17	66.7% (53.7, 79.6)
5. Any GBS diagnosis and a neurologist encounter and a diagnostic procedure within 45 days of the diagnosis	32	14	69.6% (56.3, 82.9)
***Diagnosis codes in any position of inpatient claims***			
6. A GBS diagnosis in any position of an inpatient claim [14]	39	47	45.3% (34.8, 55.9)
7. A GBS diagnosis in any position of an inpatient claim and a neurologist encounter occurring within 45 days following diagnosis [14]	36	18	66.7% (54.1, 79.2)
8. A GBS diagnosis in any position of an inpatient claim and a diagnostic procedure within 45 days of the inpatient admission date [20]	>29	<11	76.7% (64.1, 89.4)
9. A GBS diagnosis in any position of an inpatient claim, a neurologist encounter, and a diagnostic procedure within 45 days of the inpatient admission date	>29	<11	77.5% (64.6, 90.4)
***Diagnosis codes in the primary position of inpatient or outpatient claims***			
10. A GBS diagnosis in the primary position of any claim [14]	36	27	57.1% (44.9, 69.4)
11. Two or more instances of a GBS diagnosis in the primary position of any claim within 30 days [26]	23	12	65.7% (50.0, 81.4)
12. A GBS diagnosis in the primary position of any claim and a neurologist encounter within 45 days of diagnosis [14]	34	19	64.2% (51.2, 77.1)
13. A GBS diagnosis in the primary position of any claim and a diagnostic procedure within 45 days of diagnosis [20]	>29	<11	80.0% (67.6, 92.4)
14. A GBS diagnosis in the primary position of any claim, a neurologist encounter, and a diagnostic procedure within 45 days of diagnosis	>29	<11	78.9% (66.0, 91.9)
***Diagnosis codes in the primary position of inpatient claims***			
15. A GBS diagnosis in the primary position of an inpatient claim [14,27]	>29	<11	79.5% (67.6, 91.5)
16. A GBS diagnosis in the primary position of an inpatient claim and a neurologist encounter occurring within 45 days of the inpatient admission date [14]	>29	<11	78.6% (66.2, 91.0)
17. A GBS diagnosis in the primary position of an inpatient claim and a diagnostic procedure within 45 days of the inpatient admission date [20]	>29	<11	81.6% (69.3, 93.9)
18. A GBS diagnosis in the primary position of an inpatient claim, a neurologist encounter, and a diagnostic procedure within 45 days of the inpatient admission date	>29	<11	80.6% (67.6, 93.5)

Cell sizes <11 have been suppressed according to CMS policy.

We also tested the algorithms with different prevalence assumptions for patients with electronic health records for GBS but no associated claims (Supplemental Tables 1–3). After ranking and summing each algorithm's performance on sensitivity, specificity, PPV, and NPV, we identified algorithm 15 as consistently having the lowest total score, and therefore as being the top performing algorithm regardless of the prevalence assumption used (Supplemental Table 4). Table 3 shows the relative rank-summed values for the four measures, which was calculated by dividing each rank-sum by the best algorithm's rank-sum. We found algorithms 13 and 17 were also consistently high-performing across assumptions. Beyond that, the other high-performing algorithms were conditional on the assumptions. The appendix shows individual rankings for each measure across algorithms and assumptions. In order to comply with the Medicare cell size policy, we were unable to report the specific values for each measure under each assumption.

**Table 3:**
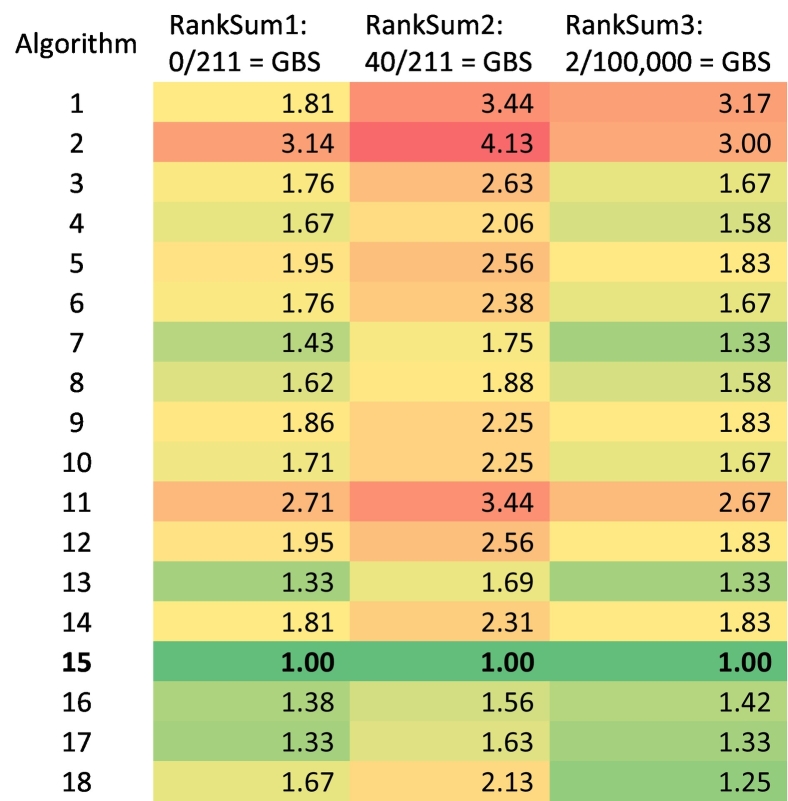
Relative Rank-Sum for algorithms under different false negative prevalence assumptions^a^.

a Red cells indicate worse-performing algorithms and green cells indicate better-performing algorithms; values are the rank-sum numbers (Supplemental Table 4) divided by the rank-sum of the best performing algorithm for each assumption (Algorithm 15).

## Discussion

This study assessed a variety of different algorithms for identifying incident cases of GBS in Medicare using chart review as the gold standard. We found that algorithm 17 had the highest PPV. As algorithms became more restrictive, the PPV tended to increase, but so did the number of false negatives. Moving from a GBS diagnosis in any position on any claim (i.e., inpatient or outpatient) to requiring a GBS diagnosis in the primary position of any claim changed the PPV from 19.0% to 57.1%. When we required the GBS diagnosis to occur on an inpatient claim, the PPV went from 45.3% in any position to 79.5% when it was in the primary position. Algorithms containing requirements for a neurologist visit or a diagnostic procedure had relatively high PPVs regardless of the position or source of the GBS diagnosis code. The PPVs ranged from 66.7%–81.6% and were consistently the highest PPVs in each diagnosis code group. Requiring these additional codes related to care could help eliminate claims where GBS was documented as a historical or rule-out diagnosis.

When selecting the ideal algorithm, maximizing PPV may not be the only metric of interest. When we assessed the relative impact of different false negative prevalence assumptions on algorithm performance, we found that the total rank-sum favored Algorithm 15 for all three assumptions. Therefore, researchers should consider which metrics are most important given the goals of the project. While Algorithm 17 had the highest PPV, Algorithm 15 was favored when considering measures beyond PPV. The PPV of Algorithm 15 was 79.5% (95% CI 67.6%, 91.5%), which aligns with the PPV of 71.2% (95% CI: 63.5%, 78.9%) reported by a study that used the same algorithm in Medicare claims. [15]

This study has some limitations. First, we excluded a large number of people (48.0%) because they did not have a UNCH medical record containing documentation of GBS. This is because patients are included in the dataset if they are treated at UNCH for any condition at any time while also being enrolled in any form of Medicare insurance. We required patients to be enrolled in Medicare Parts A and B and excluded patients enrolled in Medicare Advantage plans because they have bundled claims that lack the granularity needed for this study. We assumed the gold standard to be having a hospitalization for GBS in the medical record. While methods exist to adjust for alloyed gold standards, [25] misclassification in our gold standard final sample is highly unlikely because that would mean a patient has a medical record for GBS hospitalization, but was not actually hospitalized for GBS. These algorithms were tested in an older population and may perform differently in younger populations. We have not reported measures beyond PPV because it would have required chart-review for non-GBS cases, which was infeasible given the rarity of GBS. While we addressed this limitation by testing several different false negative prevalence assumptions, we did so by giving equal importance to sensitivity, specificity, PPV, and NPV, rather than prioritizing certain performance metrics. We were also unable to explore strata-specific PPVs (i.e. variation in PPV by sex, age, and race) due to limited sample size. Finally, the PPV changes with the prevalence of the disease in the population, and therefore the PPVs of these algorithms could change if applied in other settings outside of Medicare fee-for-service data. [14,23]

## Conclusion

This study tested a wide range of algorithms for identifying GBS cases in Medicare fee-for-service claims data. We found that Algorithm 17, a GBS diagnosis in the primary position of an inpatient claim and a diagnostic procedure within 45 days of the inpatient admission date, had the highest PPV overall. Algorithm 15, A GBS diagnosis in the primary position of an inpatient claim, maintained a high PPV and maximized the four measures of sensitivity, specificity, PPV, and NPV across different assumptions for the prevalence of false negatives.

## Funding

This work was supported by the 10.13039/100000065National Institute of Neurological Disorders and Stroke [R21 NS119867-01A1] and the Clinical and Translational Science Award program [UL1TR002489].

## CRediT authorship contribution statement

**Samantha R. Eiffert:** Writing – original draft, Validation, Software, Methodology, Formal analysis, Data curation. **Brad Wright:** Writing – review & editing, Validation, Supervision, Project administration, Methodology, Funding acquisition, Formal analysis, Conceptualization. **Joshua Nardin:** Writing – review & editing, Validation, Investigation. **James F. Howard:** Writing – review & editing, Validation, Investigation, Funding acquisition. **Rebecca Traub:** Writing – review & editing, Validation, Supervision, Project administration, Methodology, Investigation, Funding acquisition.

## Declaration of competing interest

None.

## Data Availability

Data are available through data use agreements with the Centers for Medicare and Medicaid and the University of North Carolina.
