# Supplementary material for: Evaluating algorithms for identifying incident Guillain-Barré Syndrome in Medicare fee-for-service claims
Source: Glob Epidemiol. 2024 May 3;7:100145. doi: 10.1016/j.gloepi.2024.100145 (PMC11090889; doi:10.1016/j.gloepi.2024.100145)
Supplement: Supplementary material [file mmc1.docx]

**Appendix**

Given the infeasibility of conducting an extensive number of chart reviews for patients with no evidence of potential GBS in the Medicare claims to identify even a single false negative case, we instead conducted a series of robustness checks to assess the relative performance of our algorithms using a hypothetical sample of cases identified as non-GBS in the Medicare claims and varying our assumptions about the rate of false negatives. This allowed us to fill in cells “C” and “D” in the following table with different values to assess how each algorithm performed under the different assumptions, where A/(A+B) = positive predictive value, A/(A+C) = sensitivity, D/(B+D) = specificity, and D/(C+D) = negative predictive value.

|  |  | Determination based on chart review | |  |
| --- | --- | --- | --- | --- |
|  |  | GBS | Non-GBS | Total |
| Determination based on Medicare claims | GBS | A | B | A+B |
|  | Non-GBS | C | D | C+D |
|  | Total | A+C | B+D | A+B+C+D |

Because our robustness check does not affect cells “A” and “B”, the PPV remains unchanged across each of these 3 hypothetical scenarios. However, the sensitivity, specificity, and negative predictive values all change. Fortunately, our focus was on the relative performance of each algorithm across these metrics, rather than the absolute values. We proceeded to rank each algorithm’s performance on each of the four metrics from 1 to 18, with 1 representing the best performing algorithm and 18 representing the worst performing algorithm, with ties allowed. We then summed the ranking for each algorithm across the four metrics, such that the best possible score would equal 4 (i.e., 1 x 4) and the worst possible score would equal 72 (i.e., 18 x 4), and identified the algorithms with the best (i.e., lowest total) scores. We assumed each metric has equal weight. This approach consistently identified algorithm 15 as the top performing algorithm regardless of which of our 3 assumptions is used. Algorithms 13 and 17 were also consistently high-performing. Beyond that, the other high-performing algorithms were conditional on the assumptions.

**Assumption 1. Matched Controls (N=211), No GBS cases missed (all 211 = Non-GBS)**

Our first analysis drew a hypothetical sample of 211 individuals who had no evidence of GBS in the claims. This was done to match the 211 individuals we identified with evidence of potential GBS in the claims. Given an estimated GBS prevalence of 1-2 cases per 100,000 population, we then assumed that there were no false negatives in that hypothetical sample of individuals drawn from the claims (i.e., no true GBS cases present among those individuals whose claims had no evidence of GBS).

**Supplemental Table 1. Algorithms Ranked Best to Worst Under Assumption 1, by Metric**

| Ranking | Sensitivity | Specificity | PPV | NPV | Ranksum |
| --- | --- | --- | --- | --- | --- |
| 1 – Best | 1 | 17, 18 | 17 | 1 | 38 |
| 2 | 6 |  | 18 | 6 | 66 |
| 3 | 3 | 13, 14 | 13 | 3 | 37 |
| 4 | 7, 10 |  | 15 | 7, 10 | 35 |
| 5 |  | 9, 15, 16 | 14 |  | 41 |
| 6 | 15 |  | 16 | 15 | 37 |
| 7 | 4, 12 |  | 9 | 4 | 30 |
| 8 |  | 8 | 8 | 12 | 34 |
| 9 | 16, 8 | 11 | 5 | 8, 16 | 39 |
| 10 |  | 5 | 4, 7 |  | 36 |
| 11 | 13, 5 | 4 |  | 5, 13 | 57 |
| 12 |  | 7 | 11 |  | 41 |
| 13 | 17, 9 | 12 | 12 | 17 | 28 |
| 14 |  | 10 | 10 | 9 | 38 |
| 15 | 14 | 2 | 3 | 14 | 21 |
| 16 | 18 | 3 | 6 | 18 | 29 |
| 17 | 2 | 6 | 2 | 2 | 28 |
| 18 – Worst | 11 | 1 | 1 | 11 | 35 |

**Top Algorithms Under Assumption 1:**

1. Algorithm 15

2. Algorithm 13 & 17

4. Algorithm 16

**Assumption 2. Matched Controls (N=211), Same Rate of True GBS cases (40 of 211 = GBS)**

Our second analysis also drew a hypothetical sample of 211 individuals who had no evidence of GBS in the claims. This time, however, we assumed that the rate of true GBS cases was exactly the same in both groups (i.e., those with and without evidence of GBS in the claims). In other words, where assumption 1 assumed no false negatives, assumption 2 assumed an equal number of true positives and false negatives. It must be stressed that this is an incredibly conservative assumption designed to test the limits of the algorithms and is very unlikely to occur. The performance of each algorithm under those conditions is shown in the next table.

**Supplemental Table 2. Algorithms Ranked Best to Worst Under Assumption 2, by Metric**

| Ranking | Sensitivity | Specificity | PPV | NPV | Ranksum |
| --- | --- | --- | --- | --- | --- |
| 1 – Best | 1 | 17, 18 | 17 | 15 | 55 |
| 2 | 6 |  | 18 | 7 | 66 |
| 3 | 3 | 13, 14 | 13 | 6 | 42 |
| 4 | 7, 10 |  | 15 | 10 | 33 |
| 5 |  | 9, 15, 16 | 14 | 4, 8, 16 | 41 |
| 6 | 15 |  | 16 |  | 38 |
| 7 | 4, 12 |  | 9 |  | 28 |
| 8 |  | 8 | 8 | 3, 12 | 30 |
| 9 | 16, 8 | 11 | 5 |  | 36 |
| 10 |  | 5 | 4, 7 | 13 | 36 |
| 11 | 5, 13 | 4 |  | 5, 9, 17 | 55 |
| 12 |  | 7 | 11 |  | 41 |
| 13 | 17, 9 | 12 | 12 |  | 27 |
| 14 |  | 10 | 10 | 14 | 37 |
| 15 | 14 | 2 | 3 | 18 | 16 |
| 16 | 18 | 3 | 6 | 11 | 25 |
| 17 | 2 | 6 | 2 | 2 | 26 |
| 18 – Worst | 11 | 1 | 1 | 1 | 34 |

**Top Algorithms Under Assumption 2:**

1. Algorithm 15

2. Algorithm 16

3. Algorithm 17

4. Algorithm 13

**Assumption 3. 100,000 Controls with Incidence of 2 cases**

Finally, our third analysis relied on the high-end estimate of GBS incidence to draw a hypothetical sample of 100,000 individuals who had no evidence of GBS in the claims. Among such a sample, we assumed that 2 true GBS cases (i.e., false negatives) would be identified, while 99,998 would be true negatives. The performance of each algorithm under those conditions is shown in the next table.

**Supplemental Table 3. Algorithms Ranked Best to Worst Under Assumption 3, by Metric**

| Ranking | Sensitivity | Specificity | PPV | NPV | Ranksum |
| --- | --- | --- | --- | --- | --- |
| 1 – Best | 1 | 2 – 18 all the same | 17 | 1 – 18 all the same | 38 |
| 2 | 6 |  | 18 |  | 36 |
| 3 | 3 |  | 13 |  | 20 |
| 4 | 7, 10 |  | 15 |  | 19 |
| 5 |  |  | 14 |  | 22 |
| 6 | 15 |  | 16 |  | 20 |
| 7 | 4, 12 |  | 9 |  | 16 |
| 8 |  |  | 8 |  | 19 |
| 9 | 16, 8 |  | 5 |  | 22 |
| 10 |  |  | 4, 7 |  | 20 |
| 11 | 5, 13 |  |  |  | 32 |
| 12 |  |  | 11 |  | 22 |
| 13 | 17, 9 |  | 12 |  | 16 |
| 14 |  |  | 10 |  | 22 |
| 15 | 14 |  | 3 |  | 12 |
| 16 | 18 |  | 6 |  | 17 |
| 17 | 2 |  | 2 |  | 16 |
| 18 – Worst | 11 | 1 | 1 |  | 15 |

**Top Algorithms Under Assumption 3: (This basically discounts Specificity and NPV)**

1. Algorithm 15

2. Algorithm 18

3. Algorithm 7, 13, 17

**Supplemental Table 4. Total Rank-Sum for algorithms under different false negative prevalence assumptions**

| Algorithm | RankSum1: 0/211 = GBS | RankSum2: 40/211 = GBS | RankSum3: 2/100,00 = GBS |
| --- | --- | --- | --- |
| 1 | 38 | 55 | 38 |
| 2 | 66 | 66 | 36 |
| 3 | 37 | 42 | 20 |
| 4 | 35 | 33 | 19 |
| 5 | 41 | 41 | 22 |
| 6 | 37 | 38 | 20 |
| 7 | 30 | 28 | 16 |
| 8 | 34 | 30 | 19 |
| 9 | 39 | 36 | 22 |
| 10 | 36 | 36 | 20 |
| 11 | 57 | 55 | 32 |
| 12 | 41 | 41 | 22 |
| 13 | 28 | 27 | 16 |
| 14 | 38 | 37 | 22 |
| 15 | **21** | **16** | **12** |
| 16 | 29 | 25 | 17 |
| 17 | 28 | 26 | 16 |
| 18 | 35 | 34 | 15 |
